# Cancer-Associated Fibroblasts Modify the Response of Prostate Cancer Cells to Androgen and Anti-Androgens in Three-Dimensional Spheroid Culture

**DOI:** 10.3390/ijms17091458

**Published:** 2016-09-01

**Authors:** Theresa Eder, Anja Weber, Hannes Neuwirt, Georg Grünbacher, Christian Ploner, Helmut Klocker, Natalie Sampson, Iris E. Eder

**Affiliations:** 1 Division of Experimental Urology, Department of Urology, Medical University of Innsbruck, Anichstrasse 35, 6020 Innsbruck, Austria; theresa.eder@charite.de (T.E.); anja.weber@i-med.ac.at (A.W.); georg.gruenbacher@gmail.com (G.G.); helmut.klocker@tirol-kliniken.at (H.K.); natalie.sampson@i-med.ac.at (N.S.); 2 Translational Radio Oncology Laboratory, Department of Radio oncology and Radiotherapy, Charité University Hospital, 10117 Berlin, Germany; 3 German Cancer Research Center (DKFZ), Heidelberg and German Cancer Consortium (DKTK) Partner Site, 10117 Berlin, Germany; 4 Department of Internal Medicine IV-Nephrology and Hypertension, Medical University of Innsbruck, Anichstrasse 35, 6020 Innsbruck, Austria; hannes.neuwirt@i-med.ac.at; 5 Department of Plastic, Reconstructive & Aesthetic Surgery, Medical University of Innsbruck, Anichstrasse 35, 6020 Innsbruck, Austria; christian.ploner@tirol-kliniken.at

**Keywords:** prostate cancer, three-dimensional epithelial–stromal spheroids, cancer-associated fibroblasts, anti-androgens, E-cadherin, Akt signaling

## Abstract

Androgen receptor (AR) targeting remains the gold standard treatment for advanced prostate cancer (PCa); however, treatment resistance remains a major clinical problem. To study the therapeutic effects of clinically used anti-androgens we characterized herein a tissue-mimetic three-dimensional (3D) in vitro model whereby PCa cells were cultured alone or with PCa-associated fibroblasts (CAFs). Notably, the ratio of PCa cells to CAFs significantly increased in time in favor of the tumor cells within the spheroids strongly mimicking PCa in vivo. Despite this loss of CAFs, the stromal cells, which were not sensitive to androgen and even stimulated by the anti-androgens, significantly influenced the sensitivity of PCa cells to androgen and to the anti-androgens bicalutamide and enzalutamide. In particular, DuCaP cells lost sensitivity to enzalutamide when co-cultured with CAFs. In LAPC4/CAF and LNCaP/CAF co-culture spheroids the impact of the CAFs was less pronounced. In addition, 3D spheroids exhibited a significant increase in E-cadherin and substantial expression of vimentin in co-culture spheroids, whereas AR levels remained unchanged or even decreased. In LNCaP/CAF spheroids we further found increased Akt signaling that could be inhibited by the phosphatidyl-inositol 3 kinase (PI3K) inhibitor LY294002, thereby overcoming the anti-androgen resistance of the spheroids. Our data show that CAFs influence drug response of PCa cells with varying impact and further suggest this spheroid model is a valuable in vitro drug testing tool.

## 1. Introduction

Prostate cancer (PCa) is the most common male cancer in Western societies [1]. Whereas patients with locally confined PCa are usually treated by surgical removal of the prostate or radiation, androgen deprivation therapy (ADT) via chemical castration has become the gold standard treatment for advanced tumors (reviewed by [2]). Despite good initial response to ADT, most patients invariably recur with castration-resistant prostate cancer (CRPC) [3]. It is well accepted that the androgen receptor (AR) is a major driver of CRPC, rendering the AR a potential anti-cancer target [4]. A number of next-generation AR targeting drugs such as enzalutamide and abiraterone have been developed [5,6,7,8]. However, despite a survival benefit in men taking these novel anti-androgens [9], resistance mechanisms frequently occur and inevitably drive disease progression [9,10,11,12].

Our current knowledge regarding the mechanisms of anti-androgen resistance in PCa mostly derives from in vitro studies using AR-positive PCa cell lines such as LNCaP, LAPC4, and DuCaP. However, cells grown in standard 2D monolayer culture have a high replicative capacity and therefore different growth characteristics and metabolic features compared to human prostate tissue [13,14], which may account for their poor predictive value of drug response. To overcome these limitations, three-dimensional (3D) culture models are increasingly being used as they are thought to better reflect the properties of human tissue. However, a number of different 3D models employ extracellular matrix (ECM) or other scaffolds that significantly influence cell biology and growth, rendering interpretation and comparison of data rather problematic. Moreover, cancer cells are strongly influenced by the surrounding stroma, which not only modulates local tumor spreading and progression but also has a significant impact on therapy resistance (recently reviewed by [15]). Despite this knowledge, few studies have combined 3D co-culture of stromal and PCa cells. Thus, a detailed characterization of 3D PCa models should be undertaken for reliable interpretation of results particularly regarding drug effects and their translation to the in vivo situation. This study aimed to characterize the usefulness of 3D cultured PCa/CAF co-culture spheroids to investigate the effects of clinically used anti-androgens.

## 2. Results

### 2.1. Growth Characteristics of LNCaP, DuCaP, and LAPC4 3D Spheroids in the Absence or Presence of Cancer-Associated Fibroblasts (CAFs)

**Figure 1:**
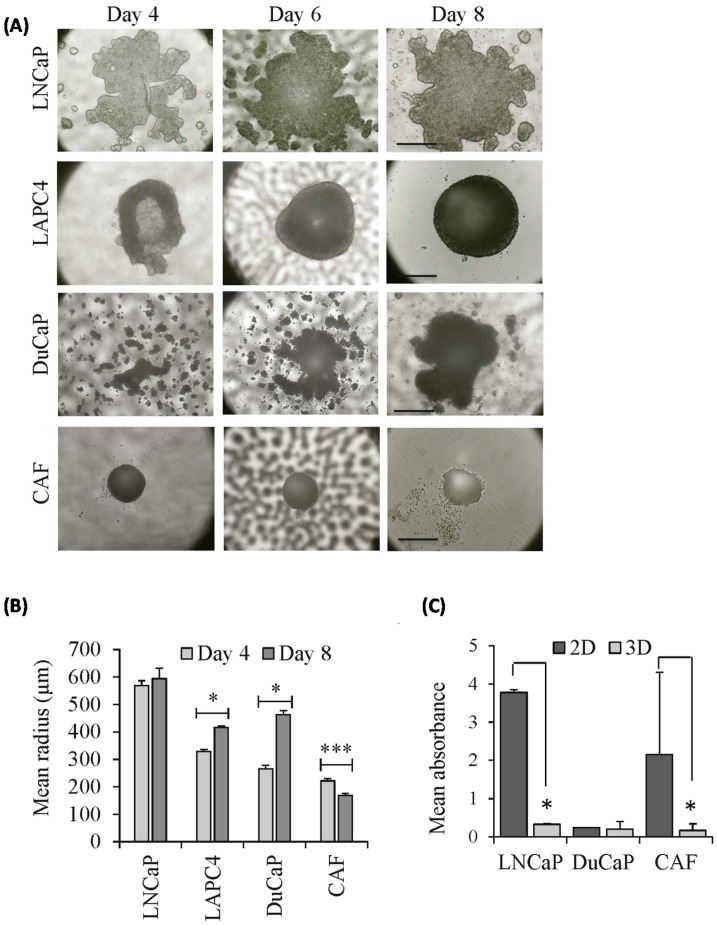
Morphology and size of monoculture spheroids established from prostate cancer (PCa) epithelial cells and PCa-associated fibroblasts (CAFs). LNCaP, LAPC4, DuCaP and CAFs were grown in scaffold-free 96-well hanging drop plates over eight days. (**A**) Representative bright-field images were taken at the day indicated (magnification 10×, scale bar: 500 μm); (**B**) mean radius of the spheroids was calculated at day 4 and day 8 as described in Materials and Methods. Error bars denote SEM. Statistical significance is shown; (**C**) Cell viability of 2D monolayer cultures and 3D spheroids was assessed by WST-1 assay, as described in Materials and Methods. Mean absorbance plus SEM was normalized to cell number. * *p* < 0.05; *** *p* < 0.001.

When cultured alone, all three PCa cell lines produced monoculture spheroids of different forms and sizes within 4–6 days. As depicted in Figure 1A, LNCaP cells produced irregular stellate spheroids with a mean radius of 594.3 ± 38.1 μm by day 8 with a moderate increase in size compared to day 4 (*p* > 0.05, Figure 1B). LAPC4 spheroids were round but compact with a mean radius of 416.8 ± 5.7 μm within eight days of culture. DuCaP cells formed compact irregular-shaped spheroids with a mean radius of 462.3 ± 14.9 μm by day 8. Notably, the formation of DuCaP spheroids was temporally delayed compared to the other cell lines. DuCaP cells formed micro-aggregates by day 4, which associated to a larger compact spheroid at day 6. Both LAPC4 and DuCaP spheroids significantly increased in size over time (Figure 1B, *p* < 0.05). CAFs cultured under 3D conditions formed compact round but very small spheroids by day 8 (mean radius of 168.4 ± 7.4 μm). Moreover, CAF spheroids did not increase in size over time but shrunk significantly instead (*p* < 0.001). Notably, however, cell viability with respect to the mitochondrial metabolic activity of CAFs and LNCaP, as assessed by WST-1 assay at day 4 of culture (Figure 1C), was significantly reduced in 3D spheroids compared to 2D monolayers. In DuCaP cells, on the other hand, 3D spheroids and 2D cultures did not significantly differ with respect to cell viability. In this cell line, absorbance values were much lower than those of LNCaP and CAF, suggesting that these cells have a lower basal metabolic activity.

We next investigated whether there are differences in population doublings (PDL) between 2D and 3D culture (Table 1). While the PDL values of LNCaP cells were lower in 3D spheroids compared to the 2D culture after four days, they reached similar levels after eight days of culture, indicating that the tumor cells need some time to adapt to 3D growth conditions. Consistent with the decreasing size of CAF 3D spheroids over time (Figure 1B), PDL calculations indicate that the number of CAFs decreased in 3D culture (Table 1).

**Figure 2:**
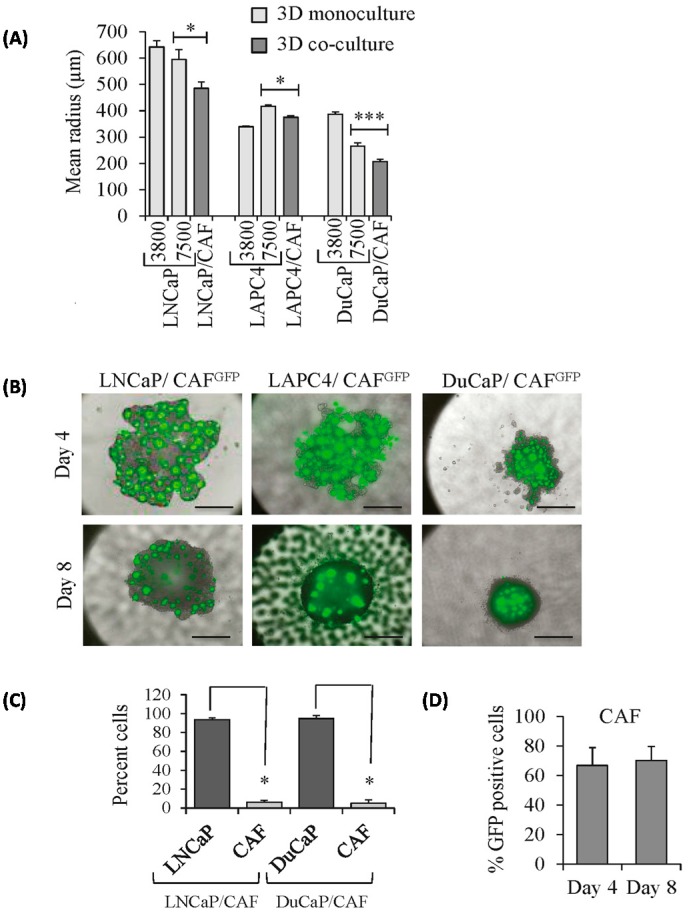
Growth characteristics of tumor epithelial–stromal co-culture spheroids. (**A**) Mean radius was determined at day 8 of culture for spheroids established from 7500 or 3800 PCa cells per drop for monocultures (LNCaP, LAPC4, DuCaP) and 3800 PCa cells and 3800 CAFs for co-culture spheroids (LNCaP/CAF, LAPC4/CAF, DuCaP/CAF); (**B**) Representative fluorescence images of co-culture spheroids after four and eight days of culture, demonstrating GFP tracked CAFs (green) as islands within the spheroids (magnification 40×, scale bar: 500 μm); (**C**) Tumor epithelial cells (LNCaP, DuCaP) and GFP-labeled CAFs were separated and quantified by FACS after eight days of co-culturing in hanging drops; (**D**) CAFs were grown as 3D spheroids over four and eight days and the percentage of GFP positive cells quantified by flow cytometry presented as mean plus SEM. * *p* < 0.05; *** *p* < 0.001.

We next investigated the characteristics of epithelial-stromal co-cultured 3D spheroids. LNCaP/CAF and DuCaP/CAF co-culture spheroids were significantly smaller than monoculture spheroids established from either 7500 or 3800 PCa cells with a mean radius of 485.7 μm (LNCaP/CAF) and 207.3 μm (DuCaP/CAF) (Figure 2A). After four days of co-culture, GFP-labeled CAFs appeared as small islands among the PCa cells (Figure 2B). Microscopic images revealed that the number of green fluorescent CAFs was reduced by day 8 compared to day 4, suggesting that the tumor cells replace the CAFs over time. Fluorescence-activated cell sorting (FACS) of 3D co-cultured spheroids into epithelial vs. stromal population in fact revealed that after eight days of co-culture less than 10% of the cells were GFP positive (Figure 2C). Specifically, LNCaP/CAF contained 6.4% ± 1.7%, DuCaP/CAF 5.2% ± 2.3% GFP-positive cells at day 8. Thus, the epithelial:stromal cell ratios were very similar between different PCa cell lines. Notably, CAFs did not significantly lose GFP expression in 3D culture over time when cultured in the absence of tumor cells since 70.2% ± 9.6% of the cells remained GFP-positive after eight days (Figure 2D).

### 2.2. E-Cadherin Protein Levels Are Increased in 3D Spheroids Compared to 2D Monolayers

**Figure 3:**
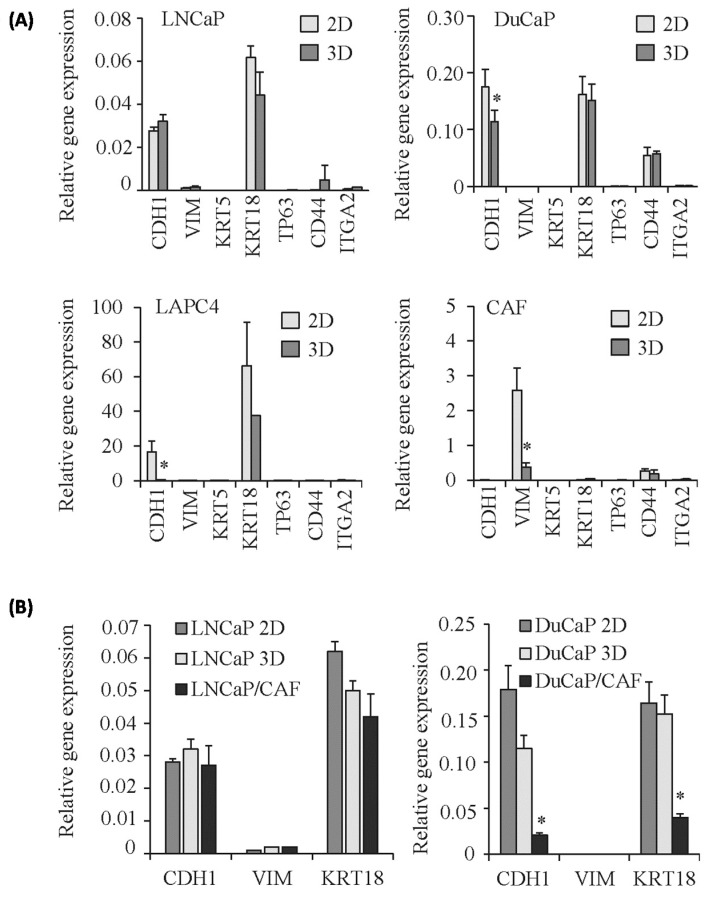
mRNA expression of cell type-specific markers in 3D spheroids compared to 2D monolayers. mRNA expression of the luminal epithelial markers E-cadherin (CDH1) and cytokeratin 18 (KER18), the stromal marker vimentin (VIM), the basal epithelial cell markers keratin 5 (KER5) and TP63, and the two cell surface glycoproteins CD44 and CD49b (ITGA2) in (**A**) 3D monoculture spheroids compared to corresponding 2D monolayer cultures and in (**B**) LNCaP/CAF and DuCaP/CAF co-culture spheroids. Target gene expression was normalized to the housekeeping genes GAPDH and HMBS and values expressed as relative gene expression plus SEM from at least three independent experiments (* different from 2D monolayer, *p* < 0.05).

3D cell culture is likely to induce fundamental changes in cell biology due to altered cell-to-cell contacts, cell polarization, and differentiation. We therefore examined the expression of the luminal epithelial markers E-cadherin (CDH1) and cytokeratin 18 (KRT18), the stromal marker vimentin (VIM), the basal epithelial markers keratin 5 (KRT5) and tumor protein P63 (TP63), and the expression of CD44 and integrin alpha 2 (ITGA2, CD49b), two cell surface glycoproteins that play an important role in cell adhesion and migration. When grown as 2D monolayers, the three PCa cell lines exhibited very similar expression patterns with high levels of CDH1 and KRT18 mRNA, but moderate expression of CD44 and ITGA2 and low to undetectable levels of VIM, KRT5, and TP63 mRNA (Figure 3A). In CAFs, on the other hand, we found a typical stromal phenotype lacking CDH1 and KRT18 expression but high levels of vimentin, which was, however, significantly decreased in 3D spheroids (*p* < 0.05). CDH1 and KRT18 mRNA expression were lower in 3D spheroids of DuCaP and LAPC4 compared to 2D cultures (*p* < 0.05). In LNCaP/CAF and DuCaP/CAF co-culture spheroids, mRNA expression of the epithelial markers CDH1 and KRT18 was further reduced compared to 3D monocultures, whereas vimentin levels remained unchanged (Figure 3B).

**Figure 4:**
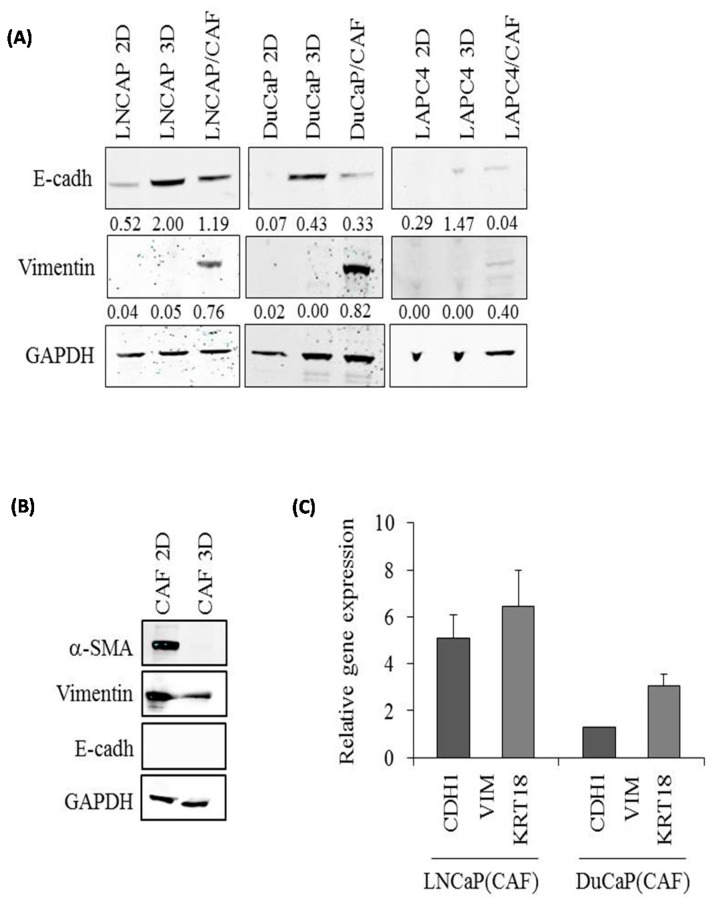
E-cadherin and vimentin protein expression in 3D spheroids compared to 2D monolayers. Expression of (**A**) E-cadherin (E-cadh) and vimentin and (**B**) α-smooth muscle actin (α-SMA), vimentin (VIM), and E-cadherin (E-cadh) was determined after eight days of culture as mono or co-culture spheroids or 2D monolayer by Western blotting. Representative blots are shown. Values denote mean densitometric intensity relative to glyceraldehyde phosphate dehydrogenase (GAPDH) from at least three independent experiments; (**C**) qPCR for E-cadherin (CDH1), vimentin (VIM) and cytokeratin 18 (KER18) in LNCaP and DuCaP cells after isolation from PCa/CAF co-culture spheroids via FACS. Values denote mean gene expression normalized against GAPDH and HMBS.

We next examined the protein expression of E-cadherin and vimentin by Western blotting. However, in contrast to mRNA expression, E-cadherin protein expression was significantly higher in 3D spheroids of all three PCa cell lines (Figure 4A) as well as in co-culture spheroids compared with 2D cultures. Vimentin, on the other hand, was undetectable in 2D culture and 3D PCa monoculture spheroids, but was detected at substantial levels in all co-culture spheroids, with highest expression in DuCaP/CAF. This high expression of vimentin was somehow unexpected due to the low to undetectable mRNA expression (Figure 3B). CAF monoculture spheroids were negative for E-cadherin but expressed high levels of vimentin and α-smooth muscle actin (α-SMA), both of which were present at significantly lower levels in 3D spheroids compared with 2D culture (Figure 4B). Notably, PCa cells were positive for E-cadherin but negative for vimentin in co-culture spheroids when analyzed after FACS separation from CAFs (Figure 4C), suggesting that vimentin expression detected in the co-culture spheroids by Western blotting (Figure 4A) originates solely from the stromal cells.

### 2.3. PCa Cells Maintain Androgen Responsiveness in 3D Culture

**Figure 5:**
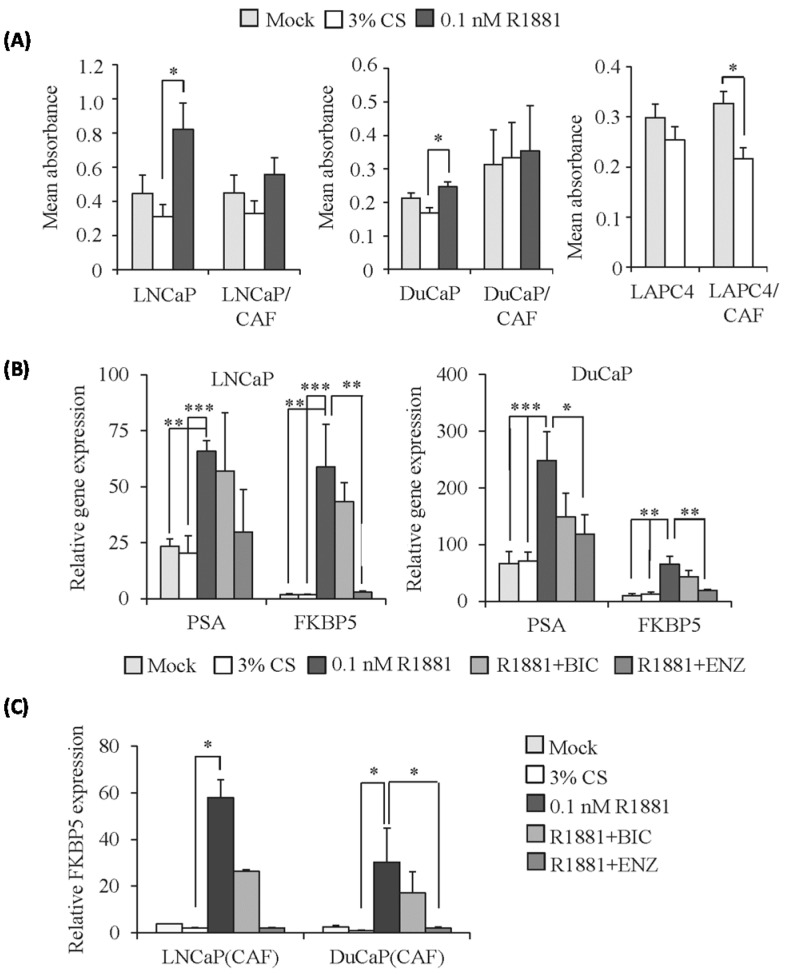
Androgen responsiveness of PCa monoculture and PCa/CAF co-culture spheroids. 3D spheroids were cultured in androgen-deprived medium containing 3% charcoal-stripped FCS (3% CS) with or without 0.1 nM R1881 and/or the anti-androgens bicalutamide (BIC) and enzalutamide (ENZ) to a final concentration of 5 μM. (**A**) Cell viability was assessed by WST-1 assay after 10 days of treatment and expressed as mean absorbance relative to vehicle control (mock) where spheroids were cultured in standard medium (10% FCS + 0.1% DMSO); (**B**) qPCR of prostate specific antigen (PSA) and FK506 binding protein 5 (FKBP5). Target gene expression was normalized to GAPDH and HMBS and expressed as relative gene expression; (**C**) FKBP5 mRNA expression was determined in GFP negative FACS sorted PCa cells from LNCaP/CAF and DuCaP/CAF co-culture spheroids following eight days of 3D culture under the conditions indicated. Values denote mean gene expression relative to the mock control with SEM (*n* = 2). * *p* < 0.05; ** *p* < 0.01; *** *p* < 0.001.

With regard to employing PCa spheroids as a tissue-mimetic model to study AR targeting agents, it is important to know if these spheroids respond to androgens and whether this might be influenced by co-culture with CAFs. To examine the androgen sensitivity of 3D spheroid cultures, we used a treatment time of 10 days. As shown in Figure 5, LNCaP spheroids displayed reduced cell viability in androgen-deprived medium containing 3% charcoal-stripped serum (3% CS, *p* > 0.05) but a significant re-stimulation of growth upon addition of 0.1 nM R1881 (3% CS + 0.1 nM R1881; *p* < 0.05) (Figure 5A). Similarly, DuCaP spheroids showed reduced cell viability in 3% CS and were significantly re-stimulated by 0.1 nM R1881 (*p* < 0.05). LAPC4 spheroids showed moderately diminished cell viability in 3% CS compared to standard medium (mock), which routinely contains 1 nM DHT.

Co-culture spheroids also showed androgen responsiveness, albeit with varying effects in the different cell lines (Figure 5A). The viability of LAPC4/CAF was significantly inhibited when maintained in 3% CS (*p* < 0.05). LNCaP/CAF also responded with reduced cell growth in 3% CS and was re-stimulated by 0.1 nM R1881, although the effect was less pronounced than in LNCaP monoculture spheroids (*p* > 0.05). DuCaP/CAF failed to respond to androgen starvation and a re-stimulation with R1881 could not be measured, indicating that CAFs mostly influence androgen responsiveness of DuCaP cells. It may be noted that the CAFs employed in this study were not androgen responsive, neither in 2D nor in 3D culture (Supplementary Materials Figure S1).

R1881 significantly upregulated expression of the androgen-regulated genes PSA and FKBP5 in LNCaP and DuCaP monoculture spheroids (Figure 5B). Moreover, the anti-androgen enzalutamide was able to abrogate this R1881-induced expression of PSA and FKBP5. Notably, this effect was also seen in LNCaP and DuCaP cells following co-culture with CAFs (Figure 5C).

### 2.4. Modified Response of PCa/CAF Co-Culture Spheroids to Anti-Androgens

**Figure 6:**
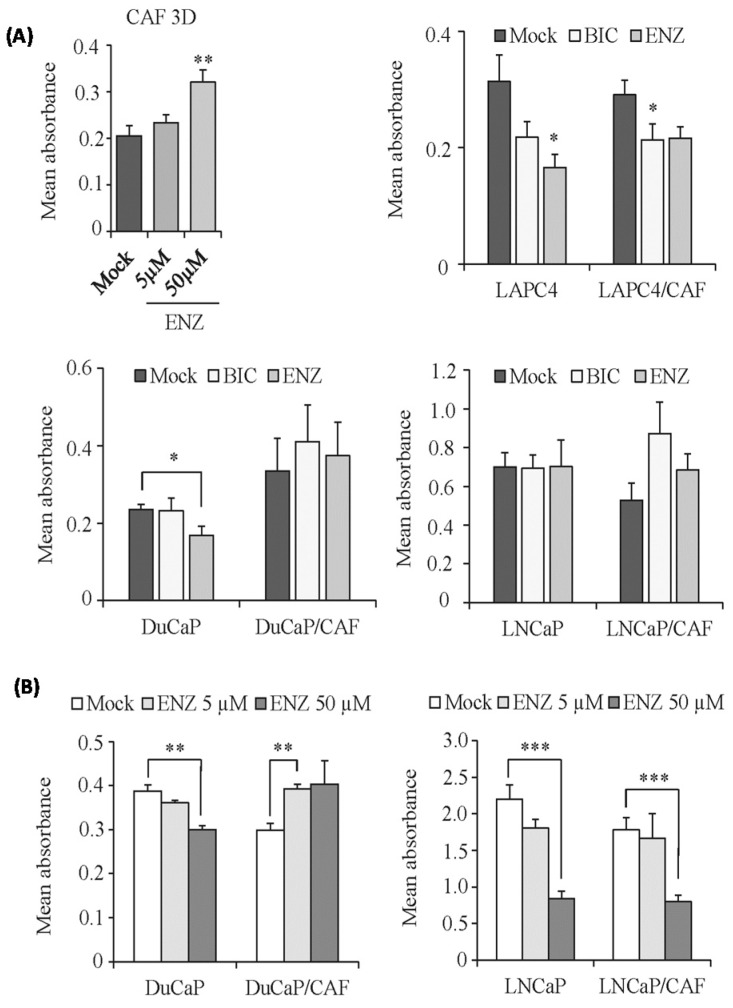
Effects of anti-androgens on prostate cancer spheroid growth. (**A**) CAF 3D spheroids were treated with 5 or 50 μM enzalutamide over 10 days. PCa monoculture and PCa/CAF co-culture spheroids were treated with bicalutamide (BIC) and enzalutamide (ENZ) at a final concentration of 5 μM in standard culture medium containing 10% FCS. Treatment was started at day 4 when spheroids were visible. Cell viability was determined after 10 days of treatment via WST-1 assay and expressed as mean absorbance; (**B**) LNCaP, DuCaP monoculture, and LNCaP/CAF, DuCaP/CAF co-culture spheroids were treated with 5 and 50 μM enzalutamide (ENZ) over 10 days and cell viability was measured via WST-1 assay. Values denote mean absorbance plus SEM (*n* = 3). * *p* < 0.05; ** *p* < 0.01; *** *p* < 0.001.

We next investigated the effect of the anti-androgens bicalutamide and enzalutamide on spheroid growth. Again we chose a treatment time of 10 days. At this time point, LNCaP cells were significantly inhibited by 5 μM enzalutamide when grown under 2D culture conditions (Supplementary Materials Figure S2). Notably, CAFs were not inhibited by the anti-androgens when cultured under 2D (Supplementary Materials Figure S2) or 3D conditions (Figure 6A). Even higher concentrations of enzalutamide (50 μM) failed to inhibit CAF 3D spheroids. This is consistent with their very low expression levels of AR (Supplementary Materials Figure S1). LAPC4 spheroids were significantly inhibited by enzalutamide (5 μM, *p* = 0.003) and to a lesser extent also by bicalutamide (5 μM, *p* > 0.05). Similar effects were observed in LAPC4/CAF co-culture spheroids. DuCaP spheroids also responded with significantly reduced cell growth upon treatment with enzalutamide (5 μM, *p* = 0.028) but not with bicalutamide (5 μM, *p* > 0.05). However, DuCaP/CAF spheroids were not inhibited by the anti-androgens at this concentration. In LNCaP monoculture and LNCaP/CAF co-culture spheroids 5 μM of bicalutamide or enzalutamide failed to reduce cell viability (Figure 6A), although this concentration of the anti-androgens inhibited LNCaP 2D monolayer cultures (Supplementary Materials Figure S2). When the concentration of enzalutamide was increased to 50 μM, however, cell viability was significantly inhibited in both LNCaP and DuCaP monoculture and LNCaP/CAF but not in DuCaP/CAF co-culture spheroids (Figure 6B), again suggesting that the impact of CAFs on anti-androgen response was most pronounced in DuCaP/CAF.

**Figure 7:**
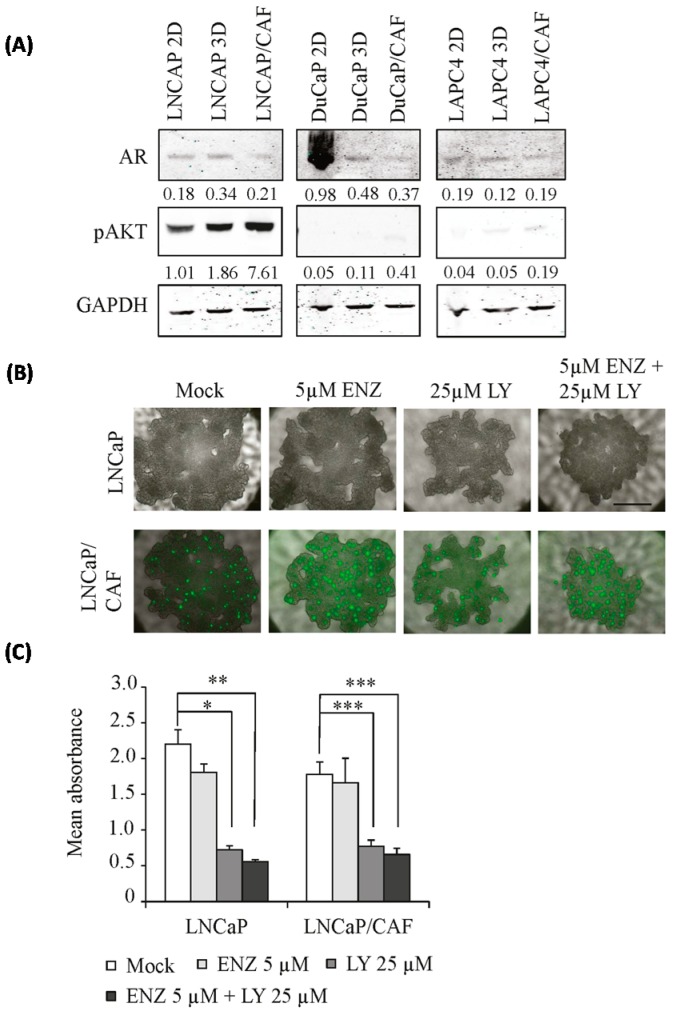
AR and pAkt expression in 3D spheroids compared to 2D monolayer cultures. (**A**) Expression of pAkt (Ser457) and androgen receptor (AR) were determined in 3D mono- and co-culture spheroids of the different cell lines by Western blotting after eight days of culture and compared with that of the corresponding 2D monolayers. GAPDH was used as normalization control. Representative blots are shown; (**B**) Representative fluorescence images showing LNCaP and LNCaP/CAF spheroids after 10 days of treatment with 5 μM enzalutamide (ENZ), 25 μM LY294002 (LY), or a combination of both (5 μM ENZ and 25 μM LY). CAFs were tracked via GFP (magnification 40×, scale bar: 500 μm); (**C**) Cell viability was assessed in LNCaP and LNCaP/CAF spheroids by WST-1 assay after 10 days under the conditions indicated and expressed as mean absorbance plus SEM. * *p* < 0.05; ** *p* < 0.01; *** *p* < 0.001.

One possible explanation for decreased sensitivity to anti-androgens, especially in DuCaP/CAF, might be elevated AR expression [4]. However, when we compared AR expression levels of 2D and 3D cultures there were no significant differences in LAPC4 and LNCaP. In DuCaP 3D spheroids, AR levels were even lower than in 2D monolayer cultures (Figure 7A). Notably, AR protein levels of DuCaP 3D spheroids were similar to those detected in LNCaP 3D spheroids, although in 2D culture, DuCaP express significantly higher levels of AR than LNCaP cells. CAFs expressed only low to undetectable levels of AR (Supplementary Materials Figure S1). Thus, the diminished response of spheroids to anti-androgens is unlikely due to elevated AR levels.

Besides AR signaling, inactivation or loss of phosphatase and tensin homologue (PTEN) with concomitant activation of Akt resulting in increased cell survival are strongly implicated in PCa progression [16]. In addition, Akt signaling has been previously associated with the formation of cell-to-cell junctions coordinated by E-cadherin [17]. We therefore looked further into the activation of Akt in LNCaP cells under 3D culture since these cells exhibit substantial amounts of pAkt (Figure 7A). Compared to 2D culture, we found that pAkt levels were increased in LNCaP monoculture and LNCaP/CAF co-culture spheroids (Figure 7A). In DuCaP and LAPC4 2D cultures, on the other hand, pAkt levels were low to undetectable and were not increased in 3D spheroids. Treatment with 25 μM of the PI3K inhibitor LY294002 significantly inhibited the growth of LNCaP and LNCaP/CAF spheroids (Figure 7B). A combination of LY294002 and 5 μM enzalutamide, however, did not result in additional growth inhibition compared to LY294002 alone.

## 3. Discussion

In the present study we used co-culture spheroids established from three PCa cell lines grown together with CAFs to investigate their usefulness as a tissue-mimetic in vitro model for PCa. The PCa cell lines used (LNCaP, DuCaP, LAPC4) all formed spheroids when grown in scaffold-free 3D hanging drops that strongly differed in morphology and size. Whereas LNCaP cells formed irregular stellate forms with moderate gain in size over time, LAPC4 and DuCaP cells produced compact round spheroids that significantly increased in size over a culture period of eight days. Notably, cell viability was generally lower in 3D culture compared to 2D monolayers and PCa/CAF co-culture spheroids were smaller than PCa monoculture spheroids. This is consistent with findings by Sieh et al., who reported that LNCaP cells grown together with human osteoblasts form smaller colonies on PEG hydrogels than LNCaP alone [18]. The reduced size of PCa/CAF co-culture spheroids compared with PCa monoculture spheroids could be explained by the lower cell viability in 3D culture in general. It is also conceivable that the cells form more compact spheroids due to the direct cell-to-cell contact of the tumor epithelial cells with the CAFs. An interesting observation was that CAFs formed small stromal “islands” within the co-culture spheroids, which decreased over time, with less than 10% CAFs remaining after eight days despite a seeding ratio between PCa cells and CAFs of 1:1. In this regard, it should be strongly considered that CAFs not only exhibited lower cell viability/metabolic activity when grown in 3D hanging drops, as assessed by WST-1 assay, but also had a reduced number of population doublings than CAFs in 2D monolayer culture. This could be simply because of suboptimal culture conditions for CAFs in the 3D hanging drops or also due to contact inhibition through the tumor cells. A loss of fibroblasts was also observed in a non-small cell lung cancer (NSCLC) co-cultured with fibroblasts in hanging drops [19]. In addition, a recent study by Chatterjee and coworkers has shown that in human PCa tissue the amount of stroma decreases with tumor progression whereas the tumor epithelial components significantly increase [20]. These data indicate that the PCa cells are the predominant cell type in human PCa in vivo and displace the stromal cells over time, a phenomenon that is similar to that observed in our PCa/CAF spheroids. Further studies are required to explore the reasons underlying the loss of stromal cells in co-culture with PCa cells.

Analysis of PCa and PCa/CAF spheroids upon expression of epithelial and stromal cell type specific markers revealed a significant increase in E-cadherin protein levels compared to 2D monolayers, suggesting that the cells strongly upregulate E-cadherin mediated cell-to-cell adhesion when grown in scaffold-free hanging drops. These results are in line with previous studies that described increased E-cadherin levels in ovarian cancer cells [14] and primary hepatocytes [21] grown under 3D conditions. Vimentin protein levels, on the other hand, remained unchanged in PCa 3D spheroids compared to 2D cultures. Substantial vimentin expression, however, was found in PCa/CAF co-culture spheroids and most likely originated solely from the CAFs, as demonstrated by separated analysis of PCa cells and CAFs following cell sorting. The relatively high levels of vimentin protein, especially in DuCaP/CAF co-culture spheroids, were somehow unexpected due to the low to undetectable mRNA expression and may be responsible for the strongly diminished anti-androgen response in this cell line compared to LNCaP and LAPC4.

AR levels were unchanged in LNCaP and LAPC4 3D spheroids, and even decreased in DuCaP spheroids and in PCa/CAF co-culture spheroids compared to 2D cultures. These changes in AR expression might also influence androgen responsiveness of the spheroids, which was reduced in LNCaP/CAF and DuCaP/CAF co-culture spheroids compared to LNCaP and DuCaP monoculture spheroids. Despite this reduced androgen sensitivity with respect to cell viability, the tumor cells within the co-culture spheroids still showed induction of androgen-regulated genes such as PSA and FKBP5 upon androgen stimulation, pointing to an essential role of the CAFs in determining the drug sensitivity of the tumor cells. Here, it should be considered that CAFs were not androgen responsive, neither in 2D nor in 3D culture; they lacked AR expression and thus were most likely not direct targets of androgens or anti-androgens themselves.

Previous studies have demonstrated that 3D cultured cells are less sensitive to anti-cancer agents than 2D cultures [22,23,24]. LNCaP cells, for instance, are more resistant to the chemotherapeutic drug docetaxel when maintained in polydimethylsiloxane (PDMS) micro-wells than conventionally grown 2D cells [23]. Ewing tumor cells grown in 3D culture also showed profound chemoresistance to a panel of cytotoxic drugs compared with adherent monolayer cultures [22]. In this study we found that the CAFs modified the sensitivity of PCa cells to the anti-androgens bicalutamide and enzalutamide; however, with different impact among the three PCa cell lines. A loss of anti-androgen sensitivity was most pronounced in DuCaP/CAF cells, where even high concentrations of enzalutamide (50 μM) failed to exert an inhibitory effect. In LNCaP/CAF, on the other hand, an increase of enzalutamide from 5 to 50 μM improved the response, whereas LAPC4/CAF still showed a response to enzalutamide even with low concentrations of 5 μM. Again, it must be considered that CAFs were not inhibited but even stimulated in growth by enzalutamide, thereby strongly influencing drug response in 3D co-culture. Increased AR expression has previously been described to promote the development of CRPC and resistance to the anti-androgen bicalutamide [4]. However, as mentioned above, AR levels remained unchanged or even decreased in PCa/CAF co-culture spheroids. Another possible reason for the increased anti-androgen resistance might be impaired access of the drug, particularly to those cells in the center of the spheroids. Although we did not observe a diminished sensitivity of 3D spheroids to other exogenously added molecules including R1881 (regulation of androgen target genes PSA and FKBP5 was very similar to that in 2D cultures), drug uptake studies are warranted to clearly answer this question.

Because of the strong upregulation of E-cadherin in the 3D spheroids herein, we further looked into pathways that could be linked to this molecule. PI3K/Akt signaling plays an important role in PCa development, progression, and treatment resistance [25,26,27,28]. Moreover, an increase in E-cadherin was associated with activation of the PI3K-Akt signaling in 3D cultured Ewing tumor cells [22]. We observed that pAkt levels were strongly increased in PTEN-negative LNCaP spheroids, with even higher levels upon co-culture with CAFs. Moreover, in contrast to 5 μM enzalutamide, which failed to inhibit spheroid growth, the PI3K inhibitor LY294002 attenuated LNCaP and even LNCaP/CAF spheroid growth, suggesting that upregulation of E-cadherin together with elevated activation of Akt is critical for growth and survival of PTEN-negative PCa spheroids, driving the cells to higher resistance towards anti-androgens.

In summary, this study shows that CAFs influence the drug response of PCa cells; however, the impact strongly varies among the different cell lines. We consider PCa/CAF co-culture spheroids a useful in vitro model with organotypic features that can be used to efficiently investigate drug effects in a more reliable tissue-mimicking situation.

## 4. Materials and Methods

### 4.1. Cell Lines and Reagents

LNCaP PCa epithelial cells, obtained from the American Type Culture Collection (ATCC, Rockville, MD, USA) and immortalized CAFs—previously established and described by Madar et al. [29]—were cultured in DMEM (Szabo Scandic, Vienna, Austria) with 10% fetal calf serum (FCS, Gibco BRL, Life Technologies, Carlsbad, CA, USA), 1% GlutaMAXTM (Gibco), and 1% penicillin and streptomycin (Lonza, Basel, Switzerland). DuCaP was obtained from Professor J. Schalken (Center for Molecular Life Science, Nijmegen, The Netherlands) and maintained in RPMI with 10% FCS [30]. LAPC4 were a gift from Professor A. Cato (Karlsruhe Institute of Technology, Karlsruhe, Germany) and maintained in RPMI 1640 (Lonza) supplemented with 10% FCS, 1% GlutaMAX™ (Gibco), 1% penicillin and streptomycin, and 1 nM dihydrotestosterone (DHT, Sigma, St. Louis, MO, USA). Cell lines’ origins and characteristics have been reviewed recently [13]. CAFs were stably transfected with green fluorescent protein (pHR-SFFV-eGFP) to differentiate them from tumor epithelial cells in co-culture spheroids using a GATEWAY-based lentiviral tetracycline-regulated conditional RNAi system as previously described [31]. All cells were cultivated at 37 °C in a humidified atmosphere with 5% CO_2_. The number of population doublings (PDL) was calculated using the formula PDL = log2 (D/D_0_) [32], with D representing the cell number at the time of harvesting and D_0_ at the time of seeding the cells.

### 4.2. 3D Cell Culture

For PCa epithelial cell or CAF monocultures, cells were seeded into 96-well Perfecta 3D hanging drop plates (Sigma) at 7500 cells per drop in 40 μL culture medium. For co-cultures, tumor cells and CAFs were mixed at a ratio of 1:1 (3800 + 3800 cells). Cells were allowed to form spheroids for four days. Culture media were replenished every 48 h. Spheroid formation and size was monitored and imaged with a JuLI Smart Fluorescence Cell Imager microscope (NanoEntek, Pleasanton, CA, USA). The mean radius (R) of the spheroids was determined according to the equation R = (1/2) × (ab)1/2 as previously described [33] using Image J software (Laboratory for Optical and Computational Instrumentation (LOCI) of the University of Wisconsin-Madison, Wisconsin-Madison, WI, USA).

### 4.3. Cell Viability Assay

Cell viability was assessed via WST-1 assay (Roche, Basel, Switzerland) according to the manufacturer’s protocol. 3D spheroids were transferred into a standard 96-well plate (Sarstedt, Germany) by centrifugation at 1200 rpm for 5 min. Before addition of WST-1 reagent, spheroids as well as 2D monolayer cultures were dissociated with 50 μL trypsin/EDTA (Lonza) at 37 °C for 20 min. Enzyme activity was stopped with 50 μL trypsin inhibitor (Sigma). Absorbance was measured with Chameleon 5025 (HVD Life Sciences, Vienna, Austria) at 420–450 nm.

### 4.4. Real-Time Quantitative PCR (qPCR)

Spheroids (*n* = 12) were collected into 1.5-mL Eppendorf tubes by floating the wells with 50 μL PBS, centrifuged at 6000 RCF for 5 min and pellets shock-frozen in liquid nitrogen. Total RNA was isolated using the RNeasy mini kit (Qiagen, Hilden, Germany). RNA concentration was measured with the NanoDrop ND-2000c (Thermo Scientific, Waltham, MA, USA). cDNA synthesis and qPCR were performed as described previously [34]. TaqMan^®^ gene expression assays for quantification of glyceraldehyde 3-phosphate dehydrogenase (GAPDH, Applied Biosystems, Foster City, CA, USA), hydroxymethylbilane synthase (HMBS, Hs00609297_m1), vimentin (VIM, Hs00185584_m1), cytokeratin 18 (KRT18, Hs01920599_gH), cytokeratin 5 (KRT5, Hs00361185_m1), E-cadherin (CDH1, Hs01013955_m1)), CD44 (CD44, Hs01075861_m1), CD49b (ITGA2, Hs00158127_m1), FKBP5 (Hs01561006_m1), and TP63 (Hs00978338_m1) were used. Primer and probe sequences of AR and PSA were as follows: AR (forward 5′-AGGATGCTCTACTTCGCCCC-3′, reverse 5′-ACTGGCTGTACATCCGGGAC-3′, probe 5′-FAM-TGGTTTTCAATGAGTACCGCATGCACA-TAMRA-3′) PSA (forward 5′-GTCTGCGGCGGTGTTCTG-3′, reverse 5′-TGCCGACCCAGCAAGATC-3′, probe: 5′-FAM-CACAGCTGCCCACTGCATCAGGA-TAMRA-3′). qPCR was carried out with ABI Prism 7500 Fast RT-PCR System (Applied Biosystems) cycler. Fold change in gene expression was determined using the mathematical model ratio $2^{-\Delta \Delta C_{t}}$ [35].

### 4.5. Cell Lysate Preparation and Western Blotting

3D spheroids (*n* = 8) were collected by floating the wells with 50 μL PBS. Samples were then centrifuged at 6000 RCF for 5 min and cell pellets shock-frozen in liquid nitrogen. Whole cell lysates from 2D and 3D cultures were generated using Tris Glycine SDS sample buffer (Gradipore, Frenchs Forest, NSW, Australia) by shaking at room temperature for 1 h and further processed via SDS-PAGE as described previously [36]. The following primary antibodies were used: α-AR (1:250; PG-21, Millipore, Darmstadt, Germany), α-vimentin (1:500, Dako, Vienna, Austria), α-pAkt (Ser475, 1:1000, Cell Signaling, Danvers, MA, USA), α-E-cadherin (1:1000, Cell Signaling), α-smooth muscle actin (SMA, 1:500, Sigma), α-GAPDH (1:50,000; Millipore).

### 4.6. Flow Cytometry 

GFP-labeled CAF spheroids (*n* = 12) were collected and digested with trypsin/EDTA as described above. Cells were re-suspended in PBS with 0.1% FCS and analyzed with a FACSCanto II flow cytometer and FACS Diva 6.1.2. and FlowJo software version 7.2.5. (BD Biosciences, Schwechat, Austria). Fixation and permeabilization of the cells was carried out with Fix and Perm^®^ Cell Fixation and Permeabilization Kit (An der Grub, Vienna, Austria). For cell sorting, co-culture spheroids (*n* = 48) were treated as described above and cells separated by fluorescent cell sorting on a FACSAria (BD Biosciences). Sorted PCa cells were directly harvested in lysis solution RL (innuPREP Micro RNA Kit, Analytik Jena, Jena, Germany).

### 4.7. Drug Treatments

To investigate the effects of drugs in 3D culture, treatment was started four days after seeding when spheroids had formed and was maintained for 10 days unless otherwise stated. To evaluate drug response in 2D culture, cells were seeded into six-well plates. Treatment started 24 h later when cells had attached to the plastic surface and maintained for 10 days unless otherwise stated. The medium was exchanged twice per week. ADT was mimicked by incubating the cells in a medium containing 3% charcoal-stripped FCS (3% CS). For androgen stimulation, the medium containing 3% CS was supplemented with R1881 as indicated. The anti-androgens bicalutamide (BIC, Astra Zeneca, Wedel, Germany) and enzalutamide (ENZ, MedChemExpress, Stockholm, Sweden) were added to a final concentration of 5 μM, unless otherwise stated, and the PI3K inhibitor LY294002 (Calbiochem, Vienna, Austria) to 25 μM in a standard culture medium.

### 4.8. Statistical Analysis

Statistical differences were calculated with the Mann–Whitney *U* test using SPSS (V15.0). Compared groups are given in the figures and/or figure legends and significances are encoded as follows: * *p* < 0.05; ** *p* < 0.01; *** *p* < 0.001. Data are presented as mean plus standard error of the mean (SEM) from three independent experiments unless otherwise stated.

## Figures and Tables

**Table 1:** Population doublings (PDL) of LNCaP and CAF in 2D and 3D culture.

Cell Line	PDL Day 4	PDL Day 8
LNCaP 2D	1.000	1.459
LNCaP 3D	0.000	1.437
CAF 2D	2.644	3.833
CAF 3D	−2.171	−3.585

## Supplementary Materials

Supplementary materials can be found at www.mdpi.com/1422-0067/17/9/1458/s1.
