# Supplementary material for: Cancer-Associated Fibroblasts Modify the Response of Prostate Cancer Cells to Androgen and Anti-Androgens in Three-Dimensional Spheroid Culture
Source: Int J Mol Sci. 2016 Sep 1;17(9):1458. doi: 10.3390/ijms17091458 (PMC5037737; doi:10.3390/ijms17091458)
Supplement: Supplementary file 1 [file ijms-17-01458-s001.pdf]

Supplementary Materials: Cancer-Associated Fibroblasts Modify the Response of Prostate Cancer Cells to Androgen and Anti-Androgens in Three-Dimensional Spheroid Culture

Theresa Eder, Anja Weber, Hannes Neuwirt, Georg Grünbacher, Christian Ploner, Helmut Klocker, Natalie Sampson and Iris E. Eder

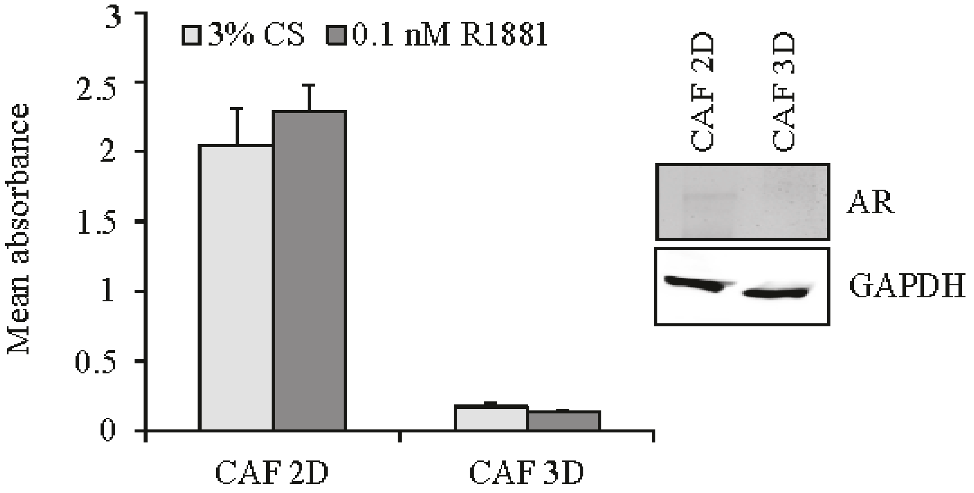

Figure S1. Androgen unresponsiveness of CAFs. CAFs were grown in a medium containing 3% CS-FCS with or without 0.1 nM R1881, either as 2D monolayers or 3D organoids. Cell viability was assessed via colorimetric WST cell viability assay and expressed as mean absorbance plus SEM. A representative blot shows AR expression in 2D and 3D cultured CAFs. GAPDH was used as a loading control.

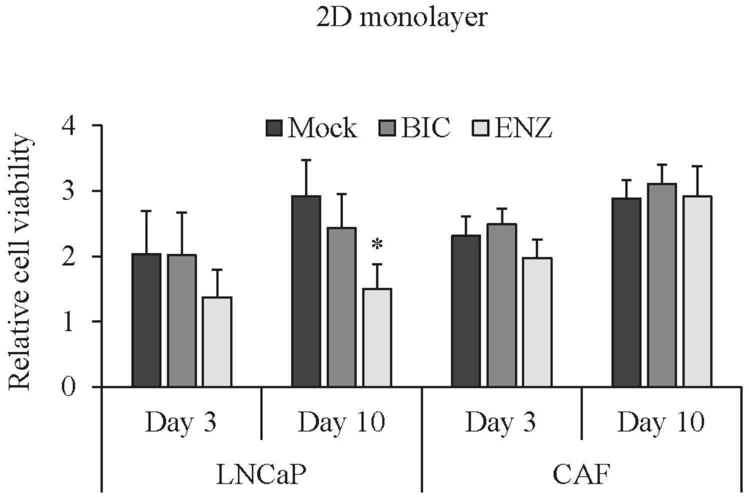

Figure S2. Effects of anti-androgens on cell viability of LNCaP and CAF grown as 2D monolayers. Cells were maintained in a standard medium (mock) with or without 5 µM of bicalutamide (BIC) or enzalutamide (ENZ). Cell viability was assessed after three and 10 days of treatment via WST-1 assay and expressed as mean absorbance plus SEM. * $p < 0.05$.
